# Reporting on one's behavior: a survey experiment on the nonvalidity of self-reported COVID-19 hygiene-relevant routine behaviors

**DOI:** 10.1017/bpp.2021.13

**Published:** 2021-03-24

**Authors:** Pelle Guldborg Hansen, Erik Gahner Larsen, Caroline Drøgemüller Gundersen

**Affiliations:** 1 Science Studies, Roskilde University, Roskilde, Denmark; 2 School of Politics and International Relations, Rutherford College, University of Kent, Canterbury, UK; 3 iNudgeyou – The Applied Behavioural Science Group, Copenhagen, Denmark

**Keywords:** behavioural public policy, anchoring, self-reports, COVID-19, behavioural methodology

## Abstract

Surveys based on self-reported hygiene-relevant routine behaviors have played a crucial role in policy responses to the COVID-19 pandemic. In this article, using anchoring to test validity in a randomized controlled survey experiment during the COVID-19 pandemic, we demonstrate that asking people to self-report on the frequency of routine behaviors are prone to significant measurement error and systematic bias. Specifically, we find that participants across age, gender, and political allegiance report higher (lower) frequencies of COVID-19-relevant behaviors when provided with a higher (lower) anchor. The results confirm that such self-reports should not be regarded as behavioral data and should primarily be used to inform policy decisions if better alternatives are not available. To this end, we discuss the use of anchoring as a validity test relative to self-reported behaviors as well as viable alternatives to self-reports when seeking to behaviorally inform policy decisions.

## Background

The sudden outbreak of the global COVID-19 pandemic has presented policy makers around the world with difficult and urgent choices to be made in the context of scientific uncertainties, painful trade-offs, and economic and logistical constraints (Sibony, 2020). As citizens’ behavior is at the heart of how the virus spreads, a central dimension for any of these choices is behavioral (Krpan *et al.*, 2021; Van Bavel *et al.*, 2020). This is true, whether regarded in terms of data to guide policy decisions, models and theories used for predicting the effects of these decisions, the nature of policy interventions, or the data obtained for evaluating the effects of the decisions. Thus, it is not surprising to find that data has played a paramount role in the response to the COVID-19 pandemic, especially data on people's behavior and actions (Betsch *et al.*, 2020).

While behavioral data, that is, data on people's actual behavior and actions, is preferable, such data is regarded as time-consuming to collect, difficult to acquire, and expensive to scale (Baumeister *et al.*, 2007). Although technological advances have made it substantially easier and less costly to obtain, especially through the use of digital software (Benartzi, 2015; Gosling & Mason, 2015; Couper, 2017), this is not the case for population-wide data in studying the types and trends of routine behaviors most relevant for combatting a pandemic like COVID-19, such as hand hygiene and social distancing.

Accordingly, it is not surprising that the traditional approach of using surveys to ask people to self-report on such behaviors has been suggested as a cheap, fast, and uncontroversial substitute for obtaining actual behavioral data to inform policy decisions in relation to the COVID-19 pandemic, for example, by the World Health Organization (WHO, 2020). Nor is it surprising that, besides an already growing body of literature using self-reported survey data to assess COVID-19 related attitudes, such as likelihood to test (Thunström *et al.*, 2020) and symptoms (Menni *et al*., 2020), several large studies have already used surveys asking people to self-report on hygiene-relevant routine behaviors. For example, to obtain data about behavior in order to inform as well as evaluate important policy decisions, such surveys have been used to examine the frequency of washing and sanitizing hands, and the frequency of social interactions (Brouard *et al.*, 2020; Harper *et al.*, 2020; HOPE, 2020; Krpan *et al.*, 2021).

Such self-reported data has also affected crucial policy decisions in the context of the COVID-19 pandemic. For example, in Denmark the HOPE project was granted DKK 27,400,000 (approximately €3,681,000) in the beginning of the pandemic to map interrelationships between the trajectory of COVID-19, policy decisions, media landscapes, and citizens behavior and well-being using, among other methods, representative surveys based on self-reported behavior (HOPE, 2020) and thus adhering to the WHO guidelines (WHO, 2020). These surveys, referred to as ‘behavioural analyses’ by the HOPE project itself (HOPE, 2020), have received massive media attention and informed crucial policy decisions by the Danish government. After the first wave of the pandemic in Denmark, for example, the surveys showed a decrease in the survey respondents self-reported frequency of washing and sanitizing their hands (from 73% to 63% reporting washing and sanitizing their hands more than 10 times a day) as well as an increase in their frequency of social interactions, which informed a decision to keep the country in lockdown (Rytgaard & Raatz, 2020). When asked by national media about the validity of self-reported answers to questions about hygiene-relevant routine behaviors, the PI defended the approach of using self-reported data by saying that ‘we know from survey research that when people respond, then it is based on a feeling, and that feeling most often has a connection with the actual behaviour… The point is that we presuppose – and in all survey research you presuppose – that there is a connection between the answers that pop up in one's head, and that which actually took place’ (*our translation*, Rytgaard & Raatz, 2020).

However, research in the behavioral sciences suggests that people often cannot report accurately, or at all, on non-salient events, including routine behaviors, such as those falling under the auspices of hygiene-relevant behaviors. Much of this research originally took place in the wake of Nisbett and Wilson's work on the unreliability of self-reports on mental processes (Nisbett & Wilson, 1977), but accelerated with work around Norbert Schwarz establishing the research area referred to as ‘cognitive aspects of survey methodology’ (Schwarz, 1999). This research area has documented how self-reports of behaviors and attitudes are strongly influenced by features of the research instrument, such as question wording, format, and context, as well as by human limitations such as poor recall when reporting on one's own behavior. As a consequence, self-reported responses may become systematically biased either because ‘the answers that pop up in one's head and what actually took place’ is so loosely connected (‘the moderate position’), or because there is nothing for the answers to connect to at all, making them mere guesses not only reflecting randomness, but irrelevant yet systematic factors (‘the strong position’).

The findings on cognitive aspects of the survey methodology have important methodological implications for the assessment of frequency reports. In particular, they show that the impact of contextual features, such as response alternatives, is more pronounced, the more poorly the behavior is represented in memory (Schwarz, 1999, p. 98). This is especially the case when behaviors are frequent and not of considerable importance (Schwarz, 1999, p. 97). Instead, when behaviors are frequent and a matter of routine, the so-called recall-and-count model rooted in folk psychology does not capture how people answer questions about behavior. Specifically, when people are asked to identify the intended behavior, search their memory for relevant episodes in a specified reference period, and count them up to arrive at a numeric answer, instances of frequent behaviors blend into generic knowledge-like representations that lack the time and space markers that allow for episodic recall (Schwarz, 1999, p. 97; see also Strube, 1987). In such cases, respondents’ answers are likely to be based on some fragmented recall and inference rules (heuristics) to compute a frequency estimate. If this is true, then asking people to self-report on the frequency of hygiene-relevant behaviors such as hand washing and social distancing may be prone to systematic biases in ways that reflect a limited, if not complete absence, of any connection to their actual behavior. Accordingly, subsequently using the self-reported data to inform policy decisions might be highly problematic.

There is, to our knowledge, no evidence on how such issues might play out in the midst of the COVID-19 pandemic. The growing body of literature on COVID-19 is to some extent beginning to pay attention to possible systematic bias in self-reported behavior. For instance, it is known that social desirability bias may affect underreporting of noncompliant behavior in surveys with sensitive questions (Krumpal, 2013). The findings relative to COVID-19 compliant behaviors are however mixed with Daoust *et al.* (2020) finding large effects, Munzert and Selb (2020) finding mixed effects, and Larsen *et al.* (2020) finding no evidence of social desirability bias.

However, the suggestion made by the field of cognitive aspects of survey methodology is far more serious than the moderate suggestion of the possible influence of more or less deliberately inflated self-reporting due to the social desirability bias. Rather, the suggestion is the stronger one that people often *cannot* report on non-salient events, including routine behaviors, such as those related to hygiene-relevant behaviors. In this perspective potential, social desirability bias is only a secondary problem. The primary problem is that self-reports of the frequency of COVID-19-compliant routine behavior is not a valid measure of the actual behaviors, but instead results from respondents applying inference rules to compute a frequency estimate. In turn, such inference rules could, if shared more generally, come to reflect various factors leading to systematic biases of modal answers based on randomness within the reasonable, rather than valid measures of behavior, such as folk psychological narratives of behavioral fatigue, survey respondents’ agreement or disagreement with the health-policies implemented to combat COVID-19, or publicly perceived trends possibly themselves informed by past surveys.

While it is not possible to check for or easily manipulate most of these inference rules experimentally, we hypothesize that anchoring frequency estimates can shed light on the validity of self-reported frequencies of behavior. There are three reasons for this. First, the influence of inference rules is more pronounced the more poorly the behavior is represented in memory. Second, anchoring is an inference rule known to robustly affect frequency judgements under uncertainty (Tversky & Kahneman, 1974; Mussweiler & Strack, 2000; Furnham & Boo, 2011). Third, anchors may easily be introduced and manipulated in the context of a survey. Thus, according to our hypothesis, while anchoring may not show what particular inference rules actually influence respondent's frequency assessments of a given behavior in representative surveys in the absence of anchoring, anchoring does test the validity of such assessments. In addition, our approach can be applied to various types of behavior in future research and thus illustrates the usefulness of anchors to examine potential challenges with measuring human behavior.

In our experiment, we use anchoring to test the validity of self-reports on the frequency of two crucial COVID-19 hygiene-relevant behaviors: (1) hand hygiene and (2) social distancing. In doing this, we replicate not only the survey questions, but also the approach of using marketing research survey providers as done by studies such as the HOPE project which informed crucial policy decisions in Denmark. This is often seen as the most reliable way to conduct survey research, in contrast to convenience samples collected by the researchers themselves, and for that reason we are confident that this case will generalize to other surveys on COVID-19 hygiene-relevant behaviors.

In addition, we also examine whether there are differences in the response to anchors across gender, age, and political partisanship. On the one hand, we do this for conventional reasons. For gender, there is a need to better understand how men and women might respond differently to the pandemic (Wenham *et al.*, 2020). For age, previous research has explored elderly people's attitudes toward COVID-19 and found no systematic differences in how young and old people respond to the pandemic (Daoust, 2020), while Bordalo *et al.* (2020) suggest that older people are less concerned about the personal health risks associated with COVID-19. For party support, the interest in social desirability bias leads to the hypothesis that government supporters might be more supportive toward government restrictions and thus more likely to answer positively to the extent to which they follow these restrictions, while the opposite should be the case for government opposition insofar that respondents’ answers involve more reflective processes.

On the other hand, by examining whether age, gender, and political partisanship moderate potential anchoring effects, we provide insights into what mechanisms mediate this effect as well as the generalizability of using anchoring as a test of the validity. In particular, if anchoring frequencies produces an anchoring effect invariably of age, gender, and especially political partisanship, it supports anchoring as a general validity test of self-reports whether this effect arises from the *anchoring-and-adjustment heuristic* (which holds that respondents adjust their boundaries of estimations according to the initial anchor presented), *confirmatory hypothesis testing* (which holds that the effect results from the activation of information that is consistent with the anchor presented), or a *general social desirability bias* (which in this context means that anchors may hint boundaries for generally sociably acceptable answers). If anchoring frequencies do not produce an anchoring effect invariably this suggests that other mechanisms are at play, such as politically or socially motivated reasoning resulting in specific social desirability bias and thus that anchoring may not serve as a general validity test in this and similar contexts.

## Method and data

### Setting

The setting of our experiment is a nationally representative survey experiment conducted in Denmark (for citizens being 18 years and older) during the global COVID-19 pandemic (*n* = 1001). The data was collected using the marketing analysis firm Gallup in the period from June 9 to June 12, 2020, and thus replicated the approach of the surveys and measures in the HOPE project to ensure comparability. This approach is state-of-the-art and adheres to the relevant WHO (2020) guidelines on how to use surveys to study behavior relative to the COVID-19 pandemic.

### Design

Different from the aforementioned surveys and WHO guidelines, respondents were sequentially randomized and asked to self-report on the frequency of the two crucial hygiene-relevant behaviors surveyed in the context of two different anchors. This was done to assess the extent to which self-reported data on the frequency of the two crucial hygiene-relevant behaviors is a valid measure in the context of the COVID-19 pandemic. The logic is that if self-reported frequencies of the targeted behaviors is a valid measure, these frequencies should not be affected by anchors. To illustrate, peoples’ answer to how many times they gave birth, bought a house, or blew up a Death Star are not likely to be affected by whether or not they are asked in the context of a low or high anchor. The same would be the case for a question relative to how many times a person has washed and sanitized his or her hands, if the respondent is able to provide a reliable and valid answer to the question according to the ‘recall-and-count’ model.

For the experiment, we provided respondents with two different treatments. The first concerns how many times the respondent has washed or sanitized hands the prior day. The second concerns how many persons the respondent had been close to (within 2 m of distance), for more than 2 minutes the prior day. Thus, our measures replicate two core questions taken from the official HOPE survey used inform important Danish policy decisions during the pandemic.

For each of the treatments, however, we first ask respondents to report their answer relative to a plausible low or high anchor. For the hand hygiene measure, the low anchor is 3 and the high anchor is 30. For the close contact measure, the low anchor is 3 and the high anchor is 15. The particular values of the anchors were chosen as realistic lower and upper boundary frequencies.

We conducted randomization tests to ensure that there were no significant differences on the pretreatment covariates. These tests found no significant differences across the groups on any of our two treatments.

### Procedure

In the survey, respondents were asked a series of nine questions. First, prior to the actual experiment, respondents answered three sociodemographic questions concerning gender, age, and party support. Next, the respondents were presented with two questions regarding each of the two hygiene-relevant behaviors, that is, frequency of washing and sanitizing their hands and the frequency of close contact. The hand hygiene issue asked how many times the respondent has washed or sanitized hands the prior day. The close contact issue asked how many persons the respondent had been within 2 m of distance, for more than 2 minutes the prior day. Again, to ensure comparability, the wordings of the questions were in all relevant aspects identical to those used in the HOPE survey. However, different from the official survey each question was preceded by the same question, but asking respondents to provide answers in terms of (more/equal/less) intervals differing between the two treatments (3/30 for the hand hygiene measure; 3/15 for the distance measure). See Supplementary Appendix A for the question wording of the measures used in the study and the questions from the HOPE project (in Danish and with the English translation).

### Analysis

Table 1 provides an overview of the sample composition. The gender split is 524 women and 477 men. The average age of the respondent is 54 years. For government support, we gave all participants in the left bloc a value of 1 (i.e., supporters of *Socialdemokraterne*, *SF*, *Radikale*, *Alternativet*, and *Enhedslisten*) and all participants in the right bloc a value of 0 (i.e., supporters of *Venstre*, *Konservative*, *Dansk Folkeparti*, *Liberal Alliance*, and *Nye Borgerlige*). We also present all differences in the outcomes for each party in Supplementary Appendix.

**Table 1.** Descriptive statistics.

Statistic	*N*	Mean	SD	Min.	Pctl(25)	Pctl(75)	Max.
Outcome: hand wash	1001	14.5	10.1	0	8	20	99
Outcome: close contact	1001	7.7	11.8	0	2	9	99
Treatment: hand wash	1001	0.5	0.5	0	0	1	1
Treatment: close contact	1001	0.5	0.5	0	0	1	1
Male	1001	0.48	0.5	0	0	1	1
Age	1001	54.0	18.5	18	39	69	90
Government supporter	884	0.59	0.49	0	0	1	1

The representative nature of the data provides substantial variation across the different groups (i.e., gender, age, and government supporters) which allows us to examine the extent to which any treatment effects differ across these groups.

We present two sets of results in the analysis. First, we examine the average treatment effects in order to explore the treatment effects for the full sample. Second, we explore heterogeneity in the effect sizes across gender, age, and political partisanship. This enables us to understand the extent to which these treatment effects generalize between groups. We present the results with simple mean differences and 95% confidence intervals (for the average treatment effects) and marginal effects from an interaction model (for the heterogeneous effects with age). All OLS regressions are available in Supplementary Appendix.

## Results

**Figure 1.**
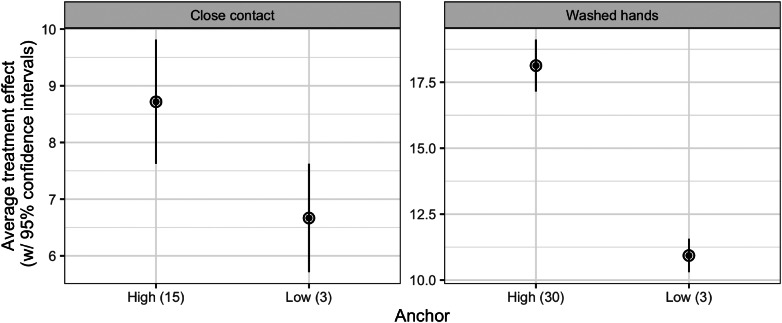
Average treatment effects. Note: Average treatment effect (with 95% confidence intervals). See Supplementary Appendix B for regression models.

Figure 1 shows the average treatment effects for the close contact and hand hygiene groups for the high and low anchors. For the close contact measure, the average response for respondents provided with the low anchor was 6.7, that is, that the respondent had been in close contact with almost seven people. In the high anchor condition, respondents reported having been in close contact with 8.7 people on average, that is, a treatment effect of 2. This effect is statistically significant (p < 0.001). Substantially, the Cohen's *d* effect size is 0.17.

For the handwashing measure, the respondents reported washing hands an average of 10.9 times when provided with the low anchor. When provided with the high anchor, we find an average treatment effect of 7.2, meaning that respondents presented with the high anchor reported washing hands 7.2 episodes more than respondents presented with the low anchor (p < 0.001). This means that people in the high anchor condition reported washing hands 18.1 times. This is a substantially large effect size (Cohen's *d* = 0.76), indicating that self-reported data for this type of behavior is effectively shaped by the type of measure.

**Figure 2.**
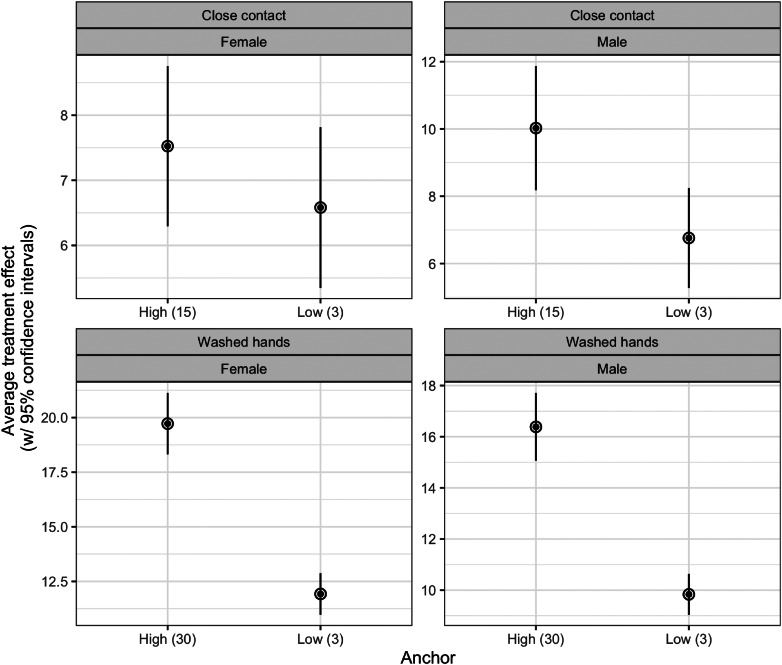
Average treatment effects for men and women. Note: Average treatment effects for men and women (with 95% confidence intervals). See Supplementary Appendix B for regression models.

Figure 2 shows the average treatment effects for men and women. We find no significant differences in the average treatment effects for gender. Specifically, the high anchor has a statistically similar treatment effect for both men and women. Formally, we tested whether there was an interaction between the gender and treatment and none of these tests were statistically significant (results available in Supplementary Appendix).

**Figure 3.**
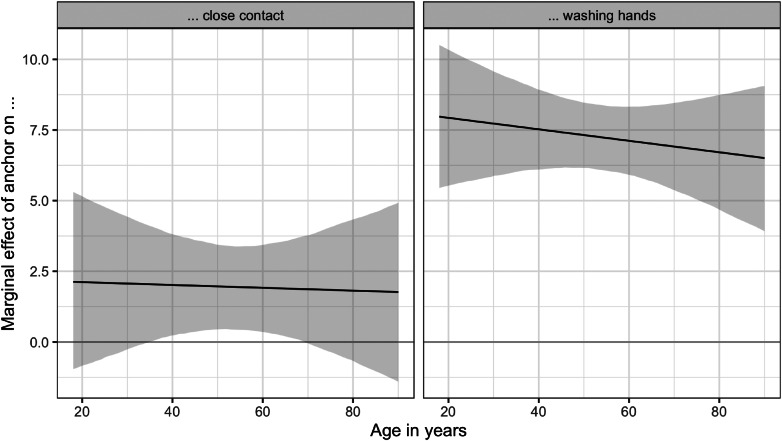
Average treatment effects across the age spectrum. Note: Marginal effect of treatment (with 95% confidence intervals). See Supplementary Appendix B for regression models.

To explore whether the treatment effects are different for different age groups, we estimated regression models where we interact the treatment with the age of the respondent. With these models, we calculated marginal average treatment effects across the age spectrum. Figure 3 reports the marginal effects of the treatment for both outcomes.

In both regressions, we find no statistically significant interaction effect. In other words, the effects of the treatments are similar for both young and older respondents. The left panel in Figure 3 shows that the marginal treatment effect of the anchor for the close contact measure is between 2 and 2.5 for all age groups. The right panel in Figure 3 shows that the marginal effect is close to seven for all age groups.

**Figure 4.**
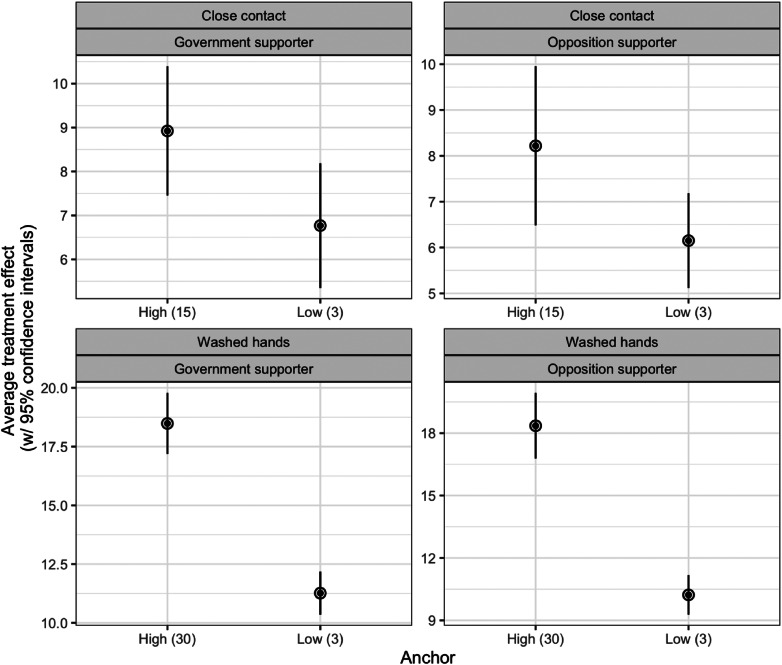
Average treatment effects for party supporters. Note: Average treatment effects for party supporters (with 95% confidence intervals). See Online Supplementary Appendix B for regression models.

Last, Figure 4 shows the average treatment effects for political partisanship. Again, we look at political partisanship to the left as government supporters (as the government in power is left-wing) and right-wing party supporters as opposition supporters. In brief, the results are consistent with the results presented above, that is, there is no evidence that these treatment effects differ across groups. In particular, we find remarkedly similar treatment effects across the two conditions for both government supporters and opposition supporters.

In sum, these results suggest that people are indeed significantly affected by anchoring when they self-report on COVID-19-related routine behaviors. The effects are present for both men and women, young and old, and government as well as opposition supporters.

## Discussion

The present experiment shows that anchoring significantly shapes self-reports of the frequency of two crucial COVID-19 hygiene-relevant behaviors – hand hygiene and social distancing – as surveyed in academic research and projects informing policy decisions relative to the pandemic.

There are three ways to respond to and interpret the results. First, that of admitting that anchoring may systematically bias otherwise valid self-reports of frequency estimates for the behaviors in question (*the folk psychological position*). However, as anchors are absent in official surveys like the HOPE survey, this position would hold that the frequency estimates provided by these surveys are not biased and hence are valid measures of actual behavior as respondents follow the ‘recall-and-count’ model intended by the survey.

Second, that of admitting that the fact that anchoring may systematically bias the self-reports of frequency estimates for the behaviors in question shows that such self-reports may be biased as well, even when such anchors are absent in the survey (*the moderate position*). That is, this position would hold that other seemingly irrelevant factors such as the social desirability or acceptability of the answers provided, for example, perceived public trends and political partisanship, could systematically bias how respondents ‘recall-and-count’ and thus such self-reports as well. The response of the moderate position would in turn be to try to control for such factors in order to estimate the ‘true value’ contained in the information signal provided by self-reported frequency estimates resulting from respondents trying to recall-and-count and otherwise trust that any remaining effects of uncertainty average out on the actual average frequencies. In fact, even if this cannot be trusted to happen, the moderate position may still hold that while self-reports of routine behaviors may have low validity, it may have high reliability, allowing a measurement of trends through longitudinal studies.

Third, by reference to Cognitive Aspects of Survey Methodology, the result supports our hypothesis that self-reports of the frequency of these behaviors as surveyed are not a valid measure of actual behavior (*the strong position*). Specifically, such effects are to be expected when the behavior is poorly represented in memory and people use fragmented recall and the application of inference rules to compute a frequency estimate. In particular, this is contrary to the ‘recall-and-count’ model assumed by the other positions that currently underpin applied research used to inform crucial policy decisions relative to the pandemic. Hence, on this position there is essentially no ‘true value’ to be salvaged in the information signal provided by self-reported frequency estimates of the behaviors in question.

So which position should one take? We argue that the strong position on the type of routine behaviors surveyed here is well established. Hence, our recommendation is to take this as the default position unless proof otherwise is provided on the validity of another position. That is, given the state-of-the-art in Cognitive Aspects of Survey Methodology, the burden of proof is on the folk psychological and moderate positions to show that there exists a link between ‘the answers that pop up in one's head and what actually took place’ relative to the routine behaviors studied, not on the strong position. Of course, if advocating the folk psychological or moderate position, one could hypothesize that an event of a global pandemic like COVID-19 might have made otherwise routine behaviors more salient and thus accessible by a recall-and-recount process. However, that does not change the burden of proof. The fact that Cognitive Aspects of Survey Methodology has found through numerous record check studies that respondents have difficulties reporting the frequencies even of major life-events (Schwarz, 2007), plus the fact that the result of the experiment reported here shows that respondents answers are so easily and invariably anchored, strongly indicates that COVID-19 has not linked the answers that pop up in one's head to what actually took place. This is in line with recent research suggesting that the pandemic has not fundamentally changed how people respond to treatments (Peyton *et al.*, 2020).

In the present experiment, respondents’ answers were significantly influenced by the anchors, but with no significant differences between gender, age-groups, and political allegiances. This further supports the hypothesis that anchoring works as a general test of validity independent of gender, age, and political partisanship. This hypothesis is consistent with the literature on the anchoring effect as conditional on uncertainty whether this effect results from the *anchoring-and-adjustment heuristic*, *confirmatory hypothesis testing*, or a *general social desirability bias*. In particular, the invariability of the effect indicates that it is caused by mechanisms not altered by politically or socially motivated reasoning. Accordingly, the invariability across sociodemographic variables not only supports the strong position but also the more general use of anchoring as a validity test of self-reported routine behaviors.

### Strengths and limitations

A strength of this experiment is that it replicates the actual approach as well as questions used to survey the frequencies of the two crucial COVID-19 hygiene-relevant behaviors targeted. That is, the experiment is nationally representative, uses one of the same market research companies (Gallup), and the same questions as those used in the Danish HOPE project, which in turn, is representative of similar international projects monitoring the crucial COVID-19 hygiene-relevant behaviors as well as adheres to the guidelines provided by WHO for gaining behavioral insights on the pandemic.

However, it should also be noted that certain limitations pertain to the experiment relevant for future research. First, the experiment *in and by itself* does not inform what inference rules actually shape frequency assessments in similar surveys where anchoring is not induced. Second, the experiment does not tell us exactly how anchoring shapes the frequency assessments made by respondents. The experiment only demonstrates that the assessment in our survey is shaped by anchoring. It is possible to argue that people actually can report the actual frequency when not influenced by anchoring (cf., the folk psychological position); or alternatively that it might be that systematic bias may be controlled for, leaving any remaining effects of uncertainty to average out on the actual average frequencies.

However, as argued, against the background of Cognitive Aspects of Survey Methodology the anchoring effects observed in our experiment are consistent with this branch of research holding that people are generally not able to report accurately, if at all, on the actual frequencies of behaviors such as those targeted here. Instead, such frequency assessments may rather merely reflect individual and collective inference rules, than systematically biased ‘true values’ that may be controlled for. In this light, our experiment does provide reasons to believe that such assessments are not just biased as a tree bend by the wind, but more like tumbleweed carried by it – and as long as evidence to the contrary is not provided, there is no reason to believe that the frequency assessments standardly surveyed are not systematically biased in a way where they merely reflect any number of irrelevant factors such as perceived trends, time-of-day effects, general social desirability bias, and the like. That is, there is currently no reason to believe that the frequency assessments usually provided (what usually pops up in one's head) is connected with actual frequencies (what actually took place). Thus, self-reports of the two COVID-19-relevant behaviors are not to be regarded as valid measures of actual behavior from a behaviorally informed point of view.

### Implications for research and policy: key guidelines for self-reported data

There are specific implications of our experiment for future work among academics and governments. We offer these in the form of guidelines about how self-reported data should be considered in relation to the study of COVID-19 hygiene-relevant behaviors; guidelines that may be extended to the study of other routine behaviors as well.

First, governments and academics should be aware of the limitations when making decisions based on self-reported data. The decision about keeping Denmark on lockdown was based on self-reported answers showing a decrease from 73% to 63% of the public reporting washing and sanitizing their hands more than 10 times a day. It affected 141,000 employees in businesses directly responsible for 2.5% of Danish BNP alone in the industry of restaurants, cafés, and bars (Rytgaard & Raatz, 2020). In our experiment, we observe a much larger ‘decrease’ from 86% (high anchor) to 52% (low anchor). For social distancing, we observe an ‘increase’ from 18.5% (low anchor) to 29.9% (high anchor). In a perspective informed by the behavioral sciences, these numbers do not necessarily reflect anything about actual behavior. Needless to say, it seems that self-reported answers could end up pointing in any direction, potentially informing other decisions impacting millions of people's health negatively. Hence, when governments and academics need to obtain evidence concerning routine behavior, we recommend that, if possible, such evidence consists in behavioral data. In particular, we suggest that policy makers consider alternative data sources and rely on self-reported data as a last resort.

Second, if self-reported data is the main source of evidence, we suggest that such work examine the validity of the measures, for example, by using anchoring to assess the variability in the compliance that can be attributed to the measurement. The experimental approach illustrates the advantage of using randomized controlled trials in the context of the COVID-19 pandemic (Haushofer & Metcalf, 2020). Only by testing the validity of our measures will we know the extent to which we are tapping into information concerning respondents’ actual behavior. Of course, and unfortunately, surveys carried out during the COVID-19 pandemic do not systematically anchor respondents’ frequency estimates to assess the validity. Yet, our experiment shows that self-reported decreases and increases might just as well reflect systematic influence of inference-rules as changes in actual behavior. In particular, while beyond the scope of this article, one possibility is that the massive media attention granted such surveys in times of a pandemic, or folk psychological mental models of ‘behavioral fatigue’ or the like promoted by the media, may themselves cause the self-reported trends.

Third, avoid using self-reported data as a substitute for behavioral data when feasible and valid alternatives for recording behavior are readily available at hand. In our experiment, we only consider the lack of validity of self-reports concerning COVID-19 hygiene-relevant routine behaviors. But few, if any policy makers and academics, would disagree to the preference for behavioral data relative to self-reported data. Hence, one might reasonably think that if behavioral data were feasible in the context of COVID-19, governments and academics would surely use such measures – and perhaps even think, that the fact that such alternative measures are not used, implies that they are not available.

This is a reasonable idea. One could argue that such alternatives are not feasible since they would have to record routine behaviors which are inherently difficult, and sometimes impossible to measure objectively given the need to track persons at all times, as well as existing privacy concerns, technological limitations, and so on. As a consequence, policy makers whether they like it or not would have to rely on self-reports in the context of the sudden outbreak of a global pandemic like COVID-19.

However, if we look at the implications of the behavioral sciences, it turns out that feasible alternatives to studying conformity levels driven by behavioral data do readily exist. Their use, though, is not so much prevented by privacy concerns or technological limitations, but rather by policy makers and researchers adopting a moderate, rather than a strong perspective, on the routine behaviors relevant to the spread of COVID-19. In this moderate perspective, behavior is primarily to be understood at the intentional level of analysis (Stanovich, 2004), that is, in terms of individuals’ beliefs, desires, attitudes, and intentions. Accordingly, monitoring COVID-19-relevant behaviors thus means monitoring individuals’ beliefs, desires, attitudes, intentions, plus their behavior – either at the level of the individual or group aggregate.

From a strong and behaviorally informed perspective, the intentional level of analysis is not the only, and often not the primary one (see, e.g., OECD, 2019). We will argue that this is especially the case when it comes to understanding the base-rates and conformity trends in routine behaviors, such as the hygiene-relevant behaviors studied relative to COVID-19. In particular, field experiments on these types of behavior clearly suggest an ‘algorithmic level’ approach, where such behaviors are better understood in terms of various cognitive processing mechanisms (e.g., attention and time available for processing information) and predicted upon specific contextual features (e.g., salience of choice options, perceived surveillance, and social information). On the strong and behavioral perspective, then, the moderate account with its emphasis on factors such as attitudes, political allegiances, gender, and age, distract decision-makers within policy and academia from the fact that behaviors such as hand hygiene seems to be more dependent upon contextual features, such as the placement and salience of sanitizers (Hansen *et al.*, 2019a) and the accompanying signage and symbolism upon entering a setting (Aarestrup *et al.*, 2016; Mobekk & Stokke, 2020; Hansen *et al.*, 2019b); and for social distancing probably more dependent upon whether you can work from home or need to take public transportation, the day of the week, whether you live in a dormitory, whether the sun was shining yesterday, the size of your family, etc.

That is not to say that the intentional level analysis does not play any role in behavior, especially in the extremes as when refraining from wearing a face-mask becomes a political statement. However, it is to say, that knowledge about variables such as individual beliefs, desires, and attitudes, are usually not of primary interest when studying routine behaviors. The object of main interest is instead of behavioral patterns as they unfold within specific contexts dependent upon various features that might seem irrelevant from the traditional perspective, but are highly relevant in a behavioral one.

It is again this background that governments and organizations like the WHO aspiring to integrate behavioral insights into policy needs to take heed of the fundamental theoretical and in turn methodological consequences that the behavioral sciences carry with them. Self-report of frequencies of routine behaviors is not a valid, nor particularly relevant, measure of actual routine behaviors – direct observation is. However, since most policy makers and scientists are far more familiar with traditional survey methodology than observational studies, we need to spell a bit out the direction this will take them.

We will briefly outline three possible directions. First of all, relative to the costs of carrying out surveys it is quite feasible to use human observers to study baseline conformity in selected crucial contexts. The survey experiment of a standard national representative survey like the one presented here amounts to the costs (approximately DKK 27,000) of having human observers measuring one-shot decision compliance levels, such as using hand sanitizer, in 25 locations for 1 day, or in one location for 25 days. In a series of recent pre-COVID-19 experiments, we used this approach to measure the percentage using the sanitizer upon entry of 40,000 visitors at a large hospital, hand hygiene and social distancing behaviors of pupils at three public schools, 5000 guests at two bars for 2 weeks, and 4500 customers entering a supermarket for 1 week. This shows how governments and behavioral scientists can observe compliance levels through longitudinal designs, and include and test interventions for improving compliance. Compliance levels from such longitudinal studies, if the context is held constant, may then in turn be used as proxies for more general trends in hand hygiene compliance levels.

Second, using existing technologies can significantly increase the number of locations as well as the time monitored. Indeed, bringing existing technologies to monitor behavior is often quite trivial and can magnify the number of possible observations substantially without infringing on privacy concerns. Thus, for example, many modern suppliers of toilet maintenance services offer and have already implemented technologies in sanitizers that not only count the number of people using, but also the number of people *not* using the equipment. In a recent experiment, we used this approach to measure baseline conformity levels and the effect of various posters and pictograms on hand wash and use of sanitizer of more than 96,000 toilet visitors at Rigshospitalet, Denmark's main hospital. Such methods and technologies for measuring the number and density of people in public places exist and in many developed countries already and are used at locations such as airports, pedestrian streets, and train stations.

Third, where direct observation is not feasible, data on routine behaviors, allowing for assembling an even more general picture of actual compliance levels and their trends exists. This may be provided by using proxies for hygiene-relevant behaviors derived from existing databases, for example, as supermarket sales numbers, cleaning service providers, mobility data, and the like, some of which the aforementioned HOPE project actually do monitor, but which have received far less public and political attention than surveys based on self-reported behaviors. The COVID-19 Community Mobility Reports from Google, for example, provides anonymized and publicly available data on movement trends over time and across regions for various categories.

While such data is of course more difficult to obtain at a nationally representative scale (as it is not a random sample that frequents, say, airports), the fact is that much routine behavior is easily measurable as soon as we leave the intentional level of analysis as the primary approach as studying routine behaviors removes the focus from individual variables to contextual ones. Hence, instead of sending out nationally representative surveys asking people to self-report on routine behaviors they cannot recall-and-count, organizations and academics should focus on feasible continuous measurements of actual behaviors seeking *the representativeness of contexts*, rather than people – and, of course, to the extent possible and permissible also collect reliable sociodemographic and intentional level data.

## Conclusion

In conclusion, self-reports on the frequency of crucial COVID-19 hygiene-relevant routine behaviors are not necessarily valid measures of actual behavioral frequencies. The findings presented here have significant implications for the current approach of using surveys as a substitute for obtaining actual behavioral data when informing policy decisions. Governments and academics should be aware of the limitations when making decisions based on self-reported data; if self-reported data is the main source of evidence, they should carefully examine the validity of the measures, for example, by using anchoring to assess the variability in the compliance that can be attributed to the measurement; and avoid using self-reported data as a substitute for behavioral data when feasible and valid alternatives for recording the latter are readily available at hand, for example, by using human observers, existing technologies, and proxies to monitor representative behaviors, rather than people.

## Supplementary material

For supplementary material accompanying this paper visit http://dx.doi.org/10.1017/bpp.2021.13. click here to view supplementary material

## Availability of data and materials

Replication material is available at the Harvard Dataverse: https://doi.org/10.7910/DVN/LAVYD9.
