# Supplementary material for: Reporting on one's behavior: a survey experiment on the nonvalidity of self-reported COVID-19 hygiene-relevant routine behaviors
Source: Behav Public Policy. 2021 Mar 24:1–18. doi: 10.1017/bpp.2021.13 (PMC8060533; doi:10.1017/bpp.2021.13)
Supplement: Supplementary file 1 [file S2398063X21000130sup001.docx]

**Online Appendix**

[A Question wording 2](#_Toc60044840)

[A.1 Danish 2](#_Toc60044841)

[A.2 English translation 2](#_Toc60044842)

[B Regression models 3](#_Toc60044843)

[C Average treatment effects: party support 7](#_Toc60044844)

# **A Question wording**

Table A.1: Question wording in Danish and English translates, our survey and HOPE

| Our survey (Danish) | Our survey (English translation) | HOPE survey (Danish) | HOPE survey (English translation) |
| --- | --- | --- | --- |
| Hvor mange gange vil du anslå, at du vaskede dine hænder eller brugte håndsprit i går? | How many times would you say you washed your hands or used sanitiser yesterday? | Hvor mange gange vil du anslå, at du vaskede dine hænder eller brugte håndsprit i går? | How many times would you say you washed your hands or used sanitiser yesterday? |
| Hvor mange gange vil du mere præcist anslå, at du vaskede dine hænder eller burgte håndsprit i går? | How many times would you more precisely say you washed your hands or used sanitiser yesterday? | - | - |
| Vi er interesseret i at vide, hvor mange personer du har været fysisk i kontakt med inden for de seneste 24 timer. Fysisk kontakt skal forstås som at være inden for 2 meters afstand af en anden person i mindst 2 minutter. | We would like to know how many people you have been in physical contact with within the last 24 hours. Physical contact is within 2 meters of another person for at least 2 minutes. | Vi er interesserede I at høre, hvor mange du har været fysisk tæt på det sidste døgn. Fysisk tæt på forstås her som tættere på end 2 meter i mindst 2 minutter. Giv os venligst dit bedste gæt. | We would like to know how many people you have been in physically near with within the last day. Physical near is within 2 meters of another person for at least 2 minutes. Please give us your best estimate. |
| Hvor mange personer vil du mere præcist anslå, at du har været fysisk i kontakt med inden for de seneste 24 timer. Fysisk kontakt forstås som at være inden for 2 meters afstand af en anden person i mindst 2 minutter. | How many people more precisely have you been in physical contact with within the last 24 hours? | - | - |

# **B Regression models**

| Table B.1: Average treatment effects | | |
| --- | --- | --- |
|  | Outcome: Handwash | Outcome: Close contact |
|  | (1) | (2) |
|  | | |
| Treatment: Handwash | 7.20^***^ |  |
|  | (0.60) |  |
|  |  |  |
| Treatment: Close contact |  | 2.05^***^ |
|  |  | (0.74) |
|  |  |  |
| Constant | 10.93^***^ | 6.67^***^ |
|  | (0.42) | (0.52) |
|  |  |  |
|  | | |
| Observations | 1,001 | 1,001 |
| R^2^ | 0.13 | 0.01 |
| Adjusted R^2^ | 0.13 | 0.01 |
| Note: Unstandardized regression coefficients with standard errors in parentheses.  ^*^ p < .1, ^**^ p < .05, ^***^ p < .01 | | |

| Table B.2: Heterogeneous effects, gender | | |
| --- | --- | --- |
|  | Outcome: Handwash | Outcome: Close contact |
|  | (1) | (2) |
|  | | |
| Treatment: Handwash | 7.80^***^ (0.82) |  |
| Treatment: Close contact |  | 0.94 (1.02) |
| Male | -2.09^**^ (0.84) | 0.18 (1.05) |
| Male * Handwash treatment | -1.25 (1.18) |  |
| Male * Close contact treatment |  | 2.32 (1.48) |
| Constant | 11.92^***^ (0.58) | 6.58^***^ (0.72) |
|  | | |
| Observations | 1,001 | 1,001 |
| R^2^ | 0.15 | 0.01 |
| Adjusted R^2^ | 0.14 | 0.01 |
| Note: Unstandardized regression coefficients with standard errors in parentheses.  ^*^ p < .1, ^**^ p < .05, ^***^ p < .01 | | |

| Table B.3: Heterogeneous effects: age | | |
| --- | --- | --- |
|  | Outcome: Handwash | Outcome: Close contact |
|  | (1) | (2) |
|  | | |
| Treatment: Handwash | 8.29^***^ (1.84) |  |
| Treatment: Close contact |  | 2.23 (2.29) |
| Age | -0.04^*^ (0.02) | -0.05^*^ (0.03) |
| Age * Handwash treatment | -0.02 (0.03) |  |
| Age * Close contact treatment |  | -0.005 (0.04) |
| Constant | 13.11^***^ (1.28) | 9.59^***^ (1.63) |
|  | | |
| Observations | 1,001 | 1,001 |
| R^2^ | 0.14 | 0.02 |
| Adjusted R^2^ | 0.13 | 0.01 |
|  | | |
| Note: Unstandardized regression coefficients with standard errors in parentheses.  ^*^ p < .1, ^**^ p < .05, ^***^ p < .01 | | |

| Table B.4: Heterogeneous effects: government supporter | | |
| --- | --- | --- |
|  | Outcome: Handwash | Outcome: Close contact |
|  | (1) | (2) |
|  | | |
| Treatment: Handwash | 8.13^***^ (0.96) |  |
| Treatment: Close contact |  | 2.07^*^ (1.16) |
| Government supporter | 1.04 (0.89) | 0.62 (1.06) |
| Government supporter * Handwash treatment | -0.91 (1.25) |  |
| Government supporter * Close contact treatment |  | 0.09 (1.51) |
| Constant | 10.23^***^ (0.70) | 6.15^***^ (0.81) |
|  | | |
| Observations | 884 | 884 |
| R^2^ | 0.15 | 0.01 |
| Adjusted R^2^ | 0.14 | 0.01 |
|  | | |
| Note: Unstandardized regression coefficients with standard errors in parentheses.  ^*^ p < .1, ^**^ p < .05, ^***^ p < .01 | | |

# **C Average treatment effects: party support**

Figure C.1: Average treatment effects, political party support
